# Venovenous extracorporeal membrane oxygenation after cardiac arrest for acute respiratory distress syndrome caused by Legionella: a case report

**DOI:** 10.1186/s13019-024-02492-6

**Published:** 2024-01-28

**Authors:** John C. Grotberg, Linda Schulte, Erin Schumer, Mary Sullivan, Kunal Kotkar, Mohammad F. Masood, Amit Pawale

**Affiliations:** 1 Division of Pulmonary and Critical Care Medicine, Washington University School of Medicine, Missouri. 660 S. Euclid Ave, St. Louis, MO 63110 USA; 2 Division of Cardiothoracic Surgery, Washington University School of Medicine, Missouri. 660 S. Euclid Ave, St. Louis, MO 63110 USA

**Keywords:** ARDS, *Legionella*, ECMO, COVID-19, Cardiac arrest, Hypoxemia

## Abstract

**Background:**

*Legionella* remains underdiagnosed in the intensive care unit and can progress to acute respiratory distress syndrome (ARDS), multiorgan failure and death. In severe cases, venovenous extracorporeal membrane oxygenation (VV-ECMO) allows time for resolution of disease with *Legionella*-targeted therapy. VV-ECMO outcomes for *Legionella* are favorable with reported survival greater than 70%. Rapid molecular polymerase chain reaction (PCR) testing of the lower respiratory tract aids in diagnosing *Legionella* with high sensitivity and specificity. We present a unique case of a patient with a positive COVID-19 test and ARDS who suffered a cardiac arrest. The patient was subsequently cannulated for VV-ECMO, and after lower respiratory tract PCR testing, *Legionella* was determined to be the cause. She was successfully treated and decannulated from VV-ECMO after eight days.

**Case presentation:**

A 53-year-old female presented with one week of dyspnea and a positive COVID-19 test. She was hypoxemic, hypotensive and had bilateral infiltrates on imaging. She received supplemental oxygen, intravenous fluids, vasopressors, broad spectrum antibiotics, and was transferred to a tertiary care center. She developed progressive hypoxemia and suffered a cardiac arrest, requiring ten minutes of CPR and endotracheal intubation to achieve return of spontaneous circulation. Despite mechanical ventilation and paralysis, she developed refractory hypoxemia and was cannulated for VV-ECMO. Dexamethasone and remdesivir were given for presumed COVID-19. Bronchoscopy with bronchoalveolar lavage (BAL) performed with PCR testing was positive for *Legionella pneumophila* and negative for COVID-19. Steroids and remdesivir were discontinued and she was treated with azithromycin. Her lung compliance improved, and she was decannulated after eight days on VV-ECMO. She was discharged home on hospital day 16 breathing room air and neurologically intact.

**Conclusions:**

This case illustrates the utility of rapid PCR testing to diagnose *Legionella* in patients with respiratory failure and the early use of VV-ECMO in patients with refractory hypoxemia secondary to *Legionella* infection. Moreover, many patients encountered in the ICU may have prior COVID-19 immunity, and though a positive COVID-19 test may be present, further investigation with lower respiratory tract PCR testing may provide alternative diagnoses. Patients with ARDS should undergo *Legionella*-specific testing, and if *Legionella* is determined to be the causative organism, early VV-ECMO should be considered in patients with refractory hypoxemia given reported high survival rates.

## Background

First identified in 1976, *Legionella pneumophila* accounts for roughly two to nine% of community acquired pneumonia cases [1]. Though incidence has increased due to improved diagnostic modalities, *Legionella* pneumonia remains underdiagnosed and underreported [2]. In severe cases, patients may develop acute respiratory distress syndrome (ARDS) requiring intensive care unit (ICU) admission. Rates of invasive mechanical ventilation (IMV) and mortality are high in patients with *Legionella* pneumonia admitted to the ICU, with rates of IMV ranging from 54.5 to 82.3% and mortality ranging from 9.1 to 41.7% [3–5]. Acute kidney injury, multiorgan failure and shock are frequently reported in patients with *Legionella* in the ICU. Failure to initiate *Legionella*-specific antibiotic therapy is associated with worse outcomes [3].

The use of venovenous extracorporeal membrane oxygenation (VV-ECMO) for severe ARDS in patients with *Legionella*, however, has shown encouraging outcomes. ECMO provides full extracorporeal support of gas exchange beyond the capabilities of IMV permitting time for diagnostic evaluation and targeted treatment. With regard to ARDS secondary to *Legionella*, the first successful case of extracorporeal life support occurred in 1989 utilizing extracorporeal CO_2_ removal with low frequency positive pressure ventilation, while the first successful use of VV-ECMO occurred in 1997 [6, 7]. Since 1989 there have been 24 published reports on the use of ECMO for ARDS caused by *Legionella*, including 73 patients in case reports, and 183 adult patients in the Extracorporeal Life Support Organization registry [8–16].

However, *Legionella* remains difficult to diagnose, and the diagnosis is more challenging when competing infectious etiologies of respiratory failure are observed. We present a case of a patient who tested positive for COVID-19 but was subsequently found to have ARDS secondary to *Legionella* using rapid molecular polymerase chain reaction (PCR) testing. The patient survived to discharge from the hospital with a good neurologic outcome after VV-ECMO cannulation post cardiac arrest.

## Case presentation

A 53-year-old female with a past medical history of rheumatoid arthritis treated with weekly methotrexate, type 2 diabetes mellitus, bipolar disorder, seizure disorder, and two previously documented COVID-19 infections (most recently documented 6 months prior) presented to the emergency department with one week of dyspnea and malaise after a trip to the Gulf Shores. She had a COVID-19 exposure and subsequently tested positive for COVID-19 in the outpatient setting three days prior to admission. In the emergency department her initial vital signs demonstrated an oxygen saturation of 72% on room air, a heart rate of 110 beats per minute, a blood pressure of 76/50 mmHg, and a temperature of 98.2 F. Her initial laboratory evaluation was notable for a white blood cell count of 13.7 10^3^/µL, serum creatinine of 2.3 m/dL, procalcitonin of 6.4 ng/mL and a lactic acid level of 8.8 mmol/L. *Legionella* urine antigen was not tested. A computed tomography scan of the chest showed diffuse bilateral consolidations concerning for multifocal pneumonia. She was resuscitated with IV fluids and a norepinephrine infusion was started for persistent hypotension and shock. She received supplemental oxygen via non-rebreather face mask and broad-spectrum antibiotics (vancomycin and piperacillin-tazobactam) were initiated. Due to the severity of her critical illness, she was transferred by helicopter to a tertiary care center for further management.

Upon landing on the helipad, she had become progressively hypoxemic, likely as a consequence of transportation at high-altitude with insufficient oxygen delivery and suffered a cardiac arrest with pulseless electrical activity. She was transported to the emergency department while receiving CPR where she was intubated. After intubation, return of spontaneous circulation was achieved. She had received ten minutes of CPR. However, her shock worsened, and she remained extremely difficult to oxygenate despite paralysis and optimizing ventilator settings. Her initial arterial blood gas showed a pH of 7.12, a P_a_CO_2_ of 42 mmHg and a P_a_O_2_ of 62 mmHg while on ventilator settings of pressure control with a driving pressure of 15 cmH_2_O, a positive end expiratory pressure (PEEP) of 15 cmH_2_O, a respiratory rate of 24 breaths per minute, and a fraction of inspired oxygen (FIO_2_) of 100%. She remained unstable over the next hour with a blood pressure of 102/61 on 0.2 mcg/kg/min norepinephrine and a heart rate of 126 beats per minute and no improvement in her oxygenation with a Murray score of 3.5. Her post-arrest neurologic status was unknown. However, given her arrest was witnessed and CPR commenced immediately and she did not appear to have an acute neurologic process on computed tomography imaging of the head, the decision was made to proceed with VV-ECMO cannulation. VV-ECMO was chosen over venoarterial ECMO (VA-ECMO) or venoarterial venous ECMO (V-AV ECMO) after a point-of-care echocardiogram demonstrated hyperdynamic left ventricular function and a mildly dilater right ventricle with evidence of right ventricular dysfunction. Her hemodynamic instability was attributed to her profound hypoxemia with right ventricular dysfunction and post-cardiac arrest vasoplegia. She was cannulated with a right femoral vein to right internal jugular vein configuration. The initial ECMO flow was 5.5 L/min, sweep gas fraction of delivered oxygen (F_d_O_2_) was 100% and a sweep gas flow was 6 L/min. She had a RESP score of 2 (risk class III) with an estimated in-hospital survival of roughly 57%. After ECMO cannulation, her oxygenation significantly improved and her ventilator settings were weaned to “rest” settings of pressure control with an inspiratory pressure of 10 cmH_2_O, PEEP of 10 cmH_2_O, respiratory rate of 10 breaths per minute, and FIO_2_ of 40%. Initial tidal volumes ranged between 60 and 75 mL corresponding to a static compliance (C_stat_) of 7.5 mL/cmH_2_O. Her arterial blood gas had improved to a pH of 7.36, a P_a_CO_2_ of 39 mmHg and a PaO2 of 81 mmHg. She was admitted to the intensive care unit with presumed COVID-19 ARDS. Her shock rapidly improved and vasopressors were discontinued with a blood pressure of 109/58 and a heart rate of 108 beats per minute. Her lactate normalized. Remdesivir and dexamethasone were initiated for presumed COVID-19 infection.

**Fig. 1:**
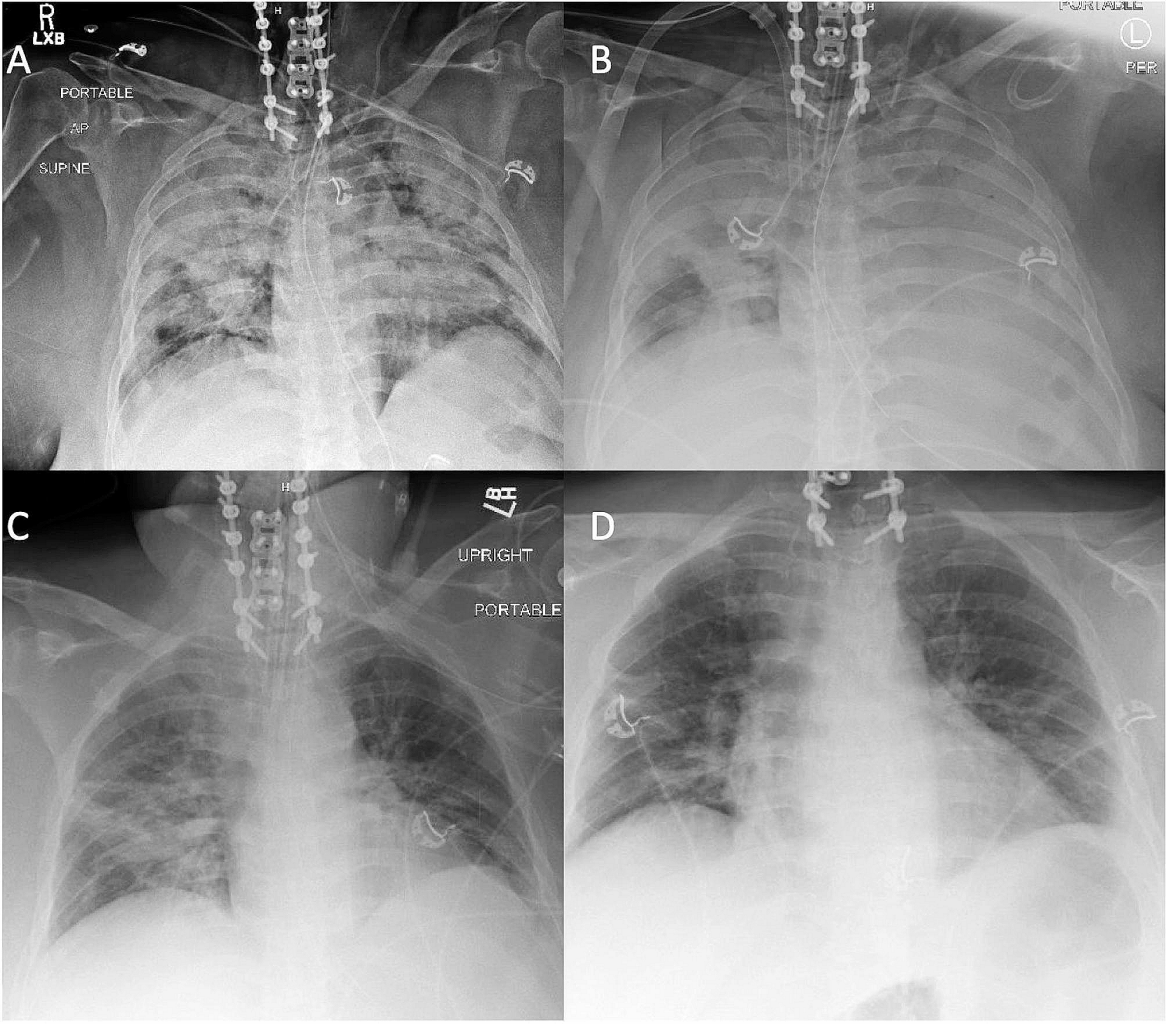
Chest x-ray on hospital day 1 after intubation and cardiac arrest. **B)** Chest x-ray on hospital day 1 after VV-ECMO cannulation. **C**) Chest x-ray on hospital day 8 after VV-ECMO decannulation. **D)** Chest x-ray on hospital day 11 after extubation

On day one the intensive care unit, a nasopharyngeal viral PCR test was performed and was negative for respiratory viruses, including COVID-19. Bronchoscopy with bronchoalveolar lavage (BAL) was then performed with rapid molecular PCR testing for viral and bacterial pathogens (BioFire® multiplex PCR, 96.2% sensitivity, 98.3% specificity). The BAL PCR test was positive for *Legionella pneumophila* and remained negative for COVID-19. Antibiotics were changed to cefepime and azithromycin 500 mg daily, and steroids were weaned off. Her respiratory system compliance improved with treatment and on day three her tidal volume had increased to 350 mL (C_stat_ 35 mL/cmH_2_O). On day five the ECMO sweep gas flow was weaned to two L/min and on day six F_d_O_2_ weaning began. On day eight her F_d_O_2_ was 21%, sweep gas flow was one L/min, and she was successfully decannulated from VV-ECMO. Her ventilator settings at the time of decannulation were pressure control with an inspiratory pressure of 15 cmH_2_O, PEEP of 10 cmH_2_O, and respiratory rate of 20 breaths per minute. C_stat_ had improved to 40 mL/cmH_2_O. On day ten she was liberated from mechanical ventilation. While on ECMO, her pulmonary infiltrates improved significantly (Fig. 1). On day 13 she was transferred out of the ICU. She completed 14 days of azithromycin and was discharged home on day 16 on room air and completely neurologically intact.

## Discussion and conclusions

This case demonstrates the benefit of rapid molecular PCR testing for the diagnosis of *Legionella* as well as the utility of early VV-ECMO for ARDS caused by *Legionella*. ECMO survival for ARDS secondary to *Legionella*, in fact, is high, ranging from 73 to 85.7% [8, 9]. This case is unique because diagnostic challenges were present, notably the concomitant positive COVID-19 test prior to admission and negative *Legionella* respiratory cultures, which most likely represented asymptomatic infection and did not impact the severity of her respiratory failure. The patient also survived with a good neurologic outcome after cannulation in the post cardiac arrest period despite having severe acute lung injury prior to her arrest. Of note, while her cardiac arrest did significantly lower her RESP score, and subsequently lowering her estimated survival, there is evidence that cardiac arrest prior to VV-ECMO cannulation is not an independent risk factor for increased mortality [17]. This case warrants timely discussion during the respiratory viral seasons as ICUs are likely to experience increased rates of COVID-19 positivity.

As previously mentioned, *Legionella* remains underdiagnosed, relying on clinical suspicion and testing availability. In a study of more than 100,000 patients in the hospital with pneumonia, only 26% were tested for *Legionella*, 1.5% of patients tested positive for *Legionella*, and of those, only 77% had received empiric *Legionella*-specific antibiotics [18]. Patients with *Legionella* were more likely to have delayed decompensation, highlighting the importance of early testing and treatment. Of note, while there is not robust pharmacokinetics data specific to azithromycin use in patients supported with ECMO, small studies suggest that minimum, maximum and area under the curve concentrations in the serum are similar when comparing ECMO to non-ECMO patients [19].

Current guidelines from the Infectious Disease Society of America (IDSA) and American Thoracic Society (ATS) suggest testing for *Legionella* by urine antigen in cases of severe community acquired pneumonia as in this patient [20]. The urine antigen test sensitivity ranges from 70 to 80% and specificity approaches 100% for *Legionella* serotype 1. However, the urine antigen test does not evaluate other serotypes [21, 22]. *Legionella* grown in culture has remained the gold standard and has 100% specificity for infection as *Legionella* does not colonize the respiratory tract. However, sensitivity ranges from 10 to 80% across studies [21, 22]. Newer rapid molecular PCR testing provides timely diagnostic evaluation of clinically relevant species and serotypes of *Legionella* with higher sensitivity (83–92%) than culture and urine antigen testing and 99.9% specificity [21, 23, 24].

In this case, the patient presented with a positive COVID-19 test in the outpatient setting, which led to initial diagnostic inertia and COVID-specific management. However, early bronchoscopy with lower respiratory tract sampling by BAL and PCR testing resulted in rapid diagnosis of *Legionella* and early treatment. The BAL fluid was also negative for COVID-19. Her initial positive test was thought to represent asymptomatic infection as she had prior immunity, and her respiratory symptoms and decompensation were solely attributed to *Legionella* infection. This is relevant as intensive care units will continue to see COVID-19 positivity in patients with prior immunity presenting, as with this patient, with alternative infections.

In conclusion, it is necessary in patients with severe respiratory failure to pursue additional diagnostic evaluation including testing for *Legionella*. While current IDSA/ATS guidelines recommend *Legionella* urine antigen testing, rapid molecular PCR testing of lower respiratory tract fluid or sputum should be used if available. Early VV-ECMO should be considered in severe cases given the favorable outcomes in this patient population.

## Data Availability

Data sharing was not applicable to this article as no datasets were generated or analyzed during this report.

## Abbreviations

ARDS: Acute Respiratory Distress Syndrome
ATS: American Thoracic Society
BAL: Bronchoalveolar Lavage
C_stat_: Static Compliance
ICU: Intensive Care Unit
IDSA: Infectious Disease Society of America
IMV: Invasive Mechanical Ventilation
PCR: Polymerase Chain Reaction
PEEP: Positive End Expiratory Pressure
VV-ECMO: Venovenous Extracorporeal Membrane Oxygenation
